# Development of the Good Health Research Practice course: ensuring quality across all health research in humans

**DOI:** 10.1186/s12961-017-0193-9

**Published:** 2017-03-31

**Authors:** Patricia Henley, Varalakshmi Elango, Olaf Horstick, Riris Andono Ahmad, Christine Maure, Pascal Launois, Corinne Merle, Jamila Nabieva, Yodi Mahendradhata

**Affiliations:** 1 London School of Hygiene and Tropical Medicine, London, United Kingdom; 2 Freelance Consultant, Chennai, India; 3 Institute of Public Health, University of Heidelberg, Heidelberg, Germany; 4 Center for Tropical Medicine, Faculty of Medicine, Universitas Gadjah Mada, Gedung PAU, Jl, Teknika Utara, Barek, Yogyakarta, 55281 Indonesia; 5 World Health Organization, Geneva, Switzerland; 6 UNICEF/UNDP/World Bank/WHO Special Programme for Research and Training in Tropical Diseases, Geneva, Switzerland; 7 Freelance Consultant, Heidelberg, Germany

**Keywords:** Capacity building, Training, Ethics, Quality, Developing Countries

## Abstract

Quality and ethics need to be embedded into all areas of research with human participants. Good Clinical Practice (GCP) guidelines are international ethical and scientific quality standards for designing, conducting, recording and reporting trials involving human participants. Compliance with GCP is expected to provide public assurance that the rights, safety and wellbeing of participants are protected and that the clinical research data are credible. However, whilst GCP guidelines, particularly their principles, are recommended across all research types, it is difficult for non-clinical trial research to fit in with the exacting requirements of GCP. There is therefore a need for guidance that allows health researchers to adhere to the principles of GCP, which will improve the quality and ethical conduct of all research involving human participants. These concerns have led to the development of the Good Health Research Practice (GHRP) course. Its goal is to ensure that research is conducted to the highest possible standards, similar to the conduct of trials to GCP. The GHRP course provides training and guidance to ensure quality and ethical conduct across all health-related research. The GHRP course has been run so far on eight occasions. Feedback from delegates has been overwhelmingly positive, with most delegates stating that the course was useful in developing their research protocols and documents. Whilst most training in research starts with a guideline, GHRP has started with a course and the experience gained over running the courses will be used to write a standardised guideline for the conduct of health-related research outside the realm of clinical trials, so that researchers, funders and ethics committees do not try to fit non-trials into clinical trials standards.

## Background

High-quality research is essential to achieving global health goals; to this end, the past few years have witnessed an expansion of health research activities in low- and middle-income countries (LMICs) [1]. There is greater demand for research institutions and scientists to efficiently organise and manage research projects, as well to meet internationally recognised standards of good practice. However, there is little training and options available to increase research capacities in the developing world [2, 3].

Of paramount concern to biomedical researchers are the rights, safety and wellbeing of research participants and should be of prime importance in the planning of any health research project. In addition, scientific integrity, including quality of the design of the project and its ethical conduct, should play a key role from the initial development of the project, through to planning, conduct and, finally, to reporting and dissemination of the results. Thus, quality and ethics need to be embedded into all areas of research with human participants.

## Currently available guidelines and their limitations

Biomedical researchers have followed specific guidelines and regulations for many years to ensure the safe and ethical conduct of their research; from the Nuremberg Code written in 1947 following the atrocities committed by the Nazis during World War II, to the World Medical Association’s Declaration of Helsinki (DoH), an international ethical standard applicable to all research on humans most recently updated in 2013 [4]. In addition to these, epidemiologists and biomedical researchers use the Council for International Organizations of Medical Sciences guidelines, based on the DoH, which focuses on ethical guidance for epidemiological studies and biomedical research involving humans [5, 6]. However, it lacks detailed guidance on how to conduct scientifically sound research and generate reliable data.

Clinical trials of investigational medicinal products have firstly benefitted from the WHO’s Guidelines for Good Clinical Practice (GCP) for trials on pharmaceutical products [7], followed by the International Council on Harmonisation Good Clinical Practice guidelines [8], both of which standardised the conduct of trials across the globe. Both GCP guidelines are international ethical and scientific quality standards for designing, conducting, recording and reporting trials involving human participants. Compliance with GCP is expected to provide public assurance that the rights, safety and wellbeing of participants are protected and that the clinical research data are credible. Countries across the globe, including LMICs, now reference these as gold standard procedures for trials. However, whilst GCP guidelines, particularly their principles, are recommended across all research types, it is difficult for non-clinical trial research, as is often the case in public health with mixed qualitative and quantitative methods, to fit in with the exacting requirements of GCP.

There is therefore a need for guidelines that allow health researchers to adhere to the principles of GCP, which will improve the quality and ethical conduct of all research involving human participants. These concerns have led to the development of the Good Health Research Practice (GHRP) course.

## Good health research practice

Although there are limitations to applying GCP to non-clinical trial research, the basic principles of ethics and quality are encouraged as a means of ensuring the protection of participants and validity of research data. Therefore, there is a need to develop principles and guidance similar to GCP that fit all types of research.

WHO/TDR (the Special Programme for Research and Training in Tropical Diseases) promotes the integration of ethics and good research practices in health research through short training courses. To this end, WHO/TDR provides support for the development of Regional Training Centres (RTCs) selected among academic and research institutions in LMICs. The goal is to establish a global network of training centres providing a portfolio of high quality training courses that collaborate and exchange experiences in the area of good practices in health research.

In 2010, WHO/TDR brought together scientists and academics from these TDR-supported RTCs across the globe to participate in the development of the GHRP training course.

The GHRP is a training programme aimed at research which falls outside the confines of clinical trials. Its goal is to ensure that research is conducted to the highest possible standards, similar to the conduct of trials to GCP. The group used their collective experience from clinical trials and conducting other health-related research, to develop an outline to ensure that all delegates would, by the end of the course, have a fully developed protocol and associated tools and procedures that would ensure quality of the conduct of the research.

The training uses a methodology based on the theory of the ‘experiential learning cycle’ and follows a ‘step-by-step learning’ approach, similar to the WHO/TDR ‘Effective project planning and evaluation’ training course. Participants apply the ethics and quality concepts and principles to their own research project, allowing them to learn by ‘doing’ and ‘reflecting’. There are short theoretical sessions followed by extensive practical sessions where participants work on their concrete experience in small groups. In the plenary, they share their observations for the benefit of all participants and projects.

A key element of the course is the discussion between researchers, as it is through participatory methods that the best possible standards for research on human health, linking quality with ethics, can be made. The outline of the course can be found in Box 1. The course has been designed to run over 4 days, with a possibility of extending this based on local requirements.

Box 1 Outline of the Good Health Research Practice training programme (to be run over 4 days)

**Table:** 

**Module 1: Introduction**
Session 1: Course introduction and overview
Session 2: Principles of research ethics and quality
Exercise 1: Risk assessment
**Module 2: Designing and planning the research**
Session 2.1: Study planning and management
Session 2.2.1: Developing the research protocol
Exercise 2: Gap analysis of protocol
Session 2.2.2: Informed consent form
Exercise 3: Review of informed consent form
Session 2.2.3: Tools for collecting data
Exercise 4: List data to be collected and draft data collection strategy
Session 2.2.4: Tools for study conduct and essential documents
Exercise 5: Identify and list key critical procedures and tools
Session 2.3: Stakeholders, study team and study sites
Exercise 6: Objectives, key steps/deliverables, work breakdown structure
Exercise 7: Organisational breakdown structure and study site checklists
Session 2.4: Research oversight
**Module 3: Conducting, recording and monitoring the research**
Session 3.1: Informed consent procedure
Exercise 8: Role-play
Session 3.2: Managing and analysing data
Session 3.3: Quality system
**Module 4: Evaluating and reporting the research**
Session 4.1: Evaluating research projects
Session 4.2: Reporting and disseminating research results
Exercise 9: Dissemination plan

## Piloting and further development

The GHRP course has been run so far on eight occasions, starting with a pilot in Heidelberg, Germany, in 2014, with over 100 delegates attending thus far. Following each facilitation of the course, the material and exercises were refined in accordance with feedback from the delegates and trainers. Full details of the development of the course, along with an analysis of the feedback provided by delegates, have been detailed in another article [9].

Response from delegates has been overwhelmingly positive, with most delegates stating that the course was useful in developing their research protocols and documents, and in obtaining peer review of their study. Delegates have so far included research project teams, Masters’ and Doctoral students, and have come to the course with outlines on a variety of different projects, from qualitative, quantitative and mixed methodology.

The piloting of the first few courses provided the facilitators with the principles of GHRP; these were developed from the key messages that were emphasised during the course (see Box 2 for the GHRP principles). These principles are introduced in the first session, and are re-visited throughout the course to ensure delegates understand how the principles integrate within each section of the course.

Box 2 Principles of good health research practice

**Table:** 

1. **Ethics and quality** underpin all types of research involving human participants
2. **Risk assessment** should be done prior to and during the course of research with appropriate mitigation measures put in place
3. **Informed consent** should be appropriate for the study and in accordance with the cultural context of the study site
4. **Procedures** should be written in line with the study protocol to ensure consistency and conformance of activities
5. **Staff qualified** through appropriate training, education and experience will undertake roles in line with their qualification
6. Study activities should be well **planned and monitored** to assure the process and data quality
7. **Privacy** of the research participants and confidentiality of all data acquired during the study should be duly protected
**8.** Research **results** and **reports** should be made publicly available

The course material was finalised in December 2015 following six pilot courses, and has been translated into Spanish and Russian. The GHRP course will expand in the future with further translations into other languages, including French. A train-the-trainer document was developed and completed in March 2016, and has been piloted twice since its inception to enable building a bank of trainers based at and around the TDR-supported RTCs. Initially, the course is open to researchers based at RTCs and their affiliated institutions. Gradually, RTCs are opening the GHRP course to a broader audience as eventually GHRP is expected to be offered more widely, in the same way that GCP is taught around the world. Currently, financial support for participation is provided from TDR, but as the course gains in popularity and demand, fees will likely be introduced in a similar fashion to GCP training. GHRP is already offered as part of the Doctoral training programme in Heidelberg, Germany, as well as in Universitas Gadjah Mada in Yogyakarta, Indonesia. There are plans also to submit to accreditation schemes for international credit recognition (e.g. European Credit Transfer and Accumulation System) to facilitate further integration into other postgraduate training programmes. This is an instrumental part of the dissemination strategy of the course.

## Conclusions

The GHRP course provides training and guidance to ensure quality and ethical conduct across all health-related research. Whilst most training in research starts with a guideline (e.g. GCP), GHRP has started with a course and the experience gained over running the courses will be used to write a standardised guideline for the conduct of health-related research outside the realm of clinical trials, so that researchers, funders and ethics committees do not try to fit non-trials into clinical trial standards. By widely disseminating the course, it is expected to generate sufficient experience and evidence that would inform the development of new pragmatic guidelines applicable to all types of human research; thus, it is hoped that GHRP becomes as ubiquitous as GCP due to its wide applicability and positive feedback received thus far.
